# Anti-malarial activity of the root extract of *Euphorbia abyssinica* (*Euphorbiaceae*) against *Plasmodium berghei* infection in mice

**DOI:** 10.1186/s12936-019-2887-7

**Published:** 2019-07-30

**Authors:** Abrham Belachew Muluye, Ashenafi Genanew Desta, Selamu Kebamo Abate, Gemechu Tiruneh Dano

**Affiliations:** 1 Department of Pharmacy, College of Medicine and Health Sciences, Bahir Dar University, Bahir Dar, Ethiopia; 2 Department of Pharmacy, College of Health Sciences, Wachamo University, Hosanna, Ethiopia; 3 Department of Medical Laboratory Sciences, College of Health Sciences, Wollega University, Nekemte, Ethiopia

**Keywords:** Anti-malarial activity, *Euphorbia abyssinica*, Malaria, Mice, *Plasmodium berghei*

## Abstract

**Background:**

More than 200 medicinal plants including *Euphorbia abyssinica* are utilized for treatment of malaria in Ethiopian traditional medical practices. However, the safety, efficacy and quality of these medicinal plants are largely unknown. Pharmacological and toxicological investigations of these plants are among the prioritized issues in every country. The aim of this study was, therefore, to evaluate the anti-malarial activity of *Euphorbia abyssinica* root extract against *Plasmodium berghei* infection in mice.

**Methods:**

The fresh roots of *Euphorbia abyssinica* were identified and collected. They were dried and extracted by 80% methanol using maceration. Acute toxicity of the extract was done using female Swiss albino mice. Anti-malarial activity of the extract was done by a standard 4-day suppressive test using chloroquine-sensitive *Plasmodium berghei*. Twenty-five male Swiss albino mice were randomly grouped into 5 groups of 5 mice each. Group I was treated with distilled water (10 ml/kg), group II, III, and IV were treated with 200, 400, and 600 mg/kg of extract, respectively and group V was treated with chloroquine (25 mg/kg). The level of parasitaemia, survival time, and variation in weight were utilized to determine the anti-malarial activity of the extract. Data was analysed using ANOVA followed by Tukey test.

**Results:**

The plant extract did not show any sign of toxicity and mortality at 2000 mg/kg. The 4-day chemosuppressive anti-malarial activities produced by the crude extract were 66.87% (P < 0.001), 84.94% (P < 0.001) and 93.69% (P < 0.001) at 200, 400 and 600 mg/kg extract, respectively, compared to distilled water treated group. Mice treated with 400 mg/kg (P < 0.01), and 600 mg/kg extract (P < 0.001) showed significant chemosuppressive anti-malarial activity variations as compared to mice treated with 200 mg/kg extract. Mice treated with 600 mg/kg extract significantly (P < 0.001) lived longer than distilled water treated mice. However, the crude extract did not cause any significant change on body weights of mice.

**Conclusions:**

From this study, it can be concluded that the root of *Euphorbia abyssinica* showed very good 4-day chemosuppressive anti-malarial activity. The plant might contain biologically active compounds which are relevant for treatment of malaria. Further phytochemical, toxicological and pharmacological investigations are, therefore, required to evaluate its anti-malarial potential.

## Background

Infectious diseases have a significant burden on global public health and economic stability. Malaria is one of the deadliest infectious diseases worldwide and the biggest health threats in Africa [1, 2]. According to the World Health Organization (WHO), about 54% and 90% of the world’s and African population are at risk of malaria, respectively. In 2017, an estimated 219 million malaria cases which caused 435,000 global deaths were reported by the WHO. Most of these cases (92%) and deaths (93%) were in Africa. Pregnant women and children under 5 years were the most vulnerable groups [3].

The emergence and spread of resistances of mosquitoes to existing insecticides and parasites to current effective anti-malarial drugs [4, 5], development of new anti-malarial drug pipelines remain sadly thin with very little chemical diversity [6], logistical problems of anti-malarial drugs in poorest malaria-endemic countries [7, 8], and lack of safe and effective anti-malarial vaccines [9] might increase the complication and resurgence of malaria in the future. As these complicating factors continue to increase, there is a great concern that malaria may continue to present a formidable challenge on the current progress made on its control and the global goal of its elimination and eradication [1]. Effective, safe, affordable and accessible alternative new anti-malarial agents and strategies are, therefore, urgently needed [10, 11].

Nearly 80% of the world’s population especially in developing countries like Ethiopia used plant-derived drugs to meet their primary healthcare demands [12]. From 14 to 28% of higher plant species are used for treatment and prevention of various health ailments in different traditional medical practices. A total of 45,000 and 7000 plant species are estimated in Africa and Ethiopia, respectively [13]. Twelve percent of Ethiopia’s floras are considered to be endemic [14, 15]. More than 1277 and 200 higher plant species are utilized for treatment and/or prevention of malaria and malarial fever in the world and in Ethiopian traditional medical practices, respectively [9, 16].

Medicinal plants of both endemic and widespread are the renewable sources for new drugs [17]. Anti-malarial drug discovery especially from plants is currently more targeted because histories proved that plants are the richest sources of effective and safe anti-malarial phytochemicals [11]. As they are many in number and contain molecules with a great variety in structure and biological activity, medicinal plants have made and continue to make a great contribution to anti-malarial drug discovery and development. However, only a limited number of medicinal plants used in traditional medical practices are investigated for their phytochemical profile, toxicological, and pharmacological activities [18, 19].

*Euphorbia abyssinica* belongs to *Euphorbiaceae,* which comprises about 300 genera and 8000 plant species*. Euphorbia* consists of more than 2000 plant species occurring in all temperate and tropical regions [20, 21]. *Euphorbia abyssinica* is a succulent, spiny tree, towering up to 10 m in height. It either stands large or grows as solitary plant in moist montane forest, humid woodlands and scrub savannah. The plant is endemic to Eastern Africa including Ethiopia, Eritrea, Somalia, and Sudan. It grows at an altitude of 1400 to 2400 metres. It has a toxic milky sap. If the sap exposed to body, it caused rash, irritant dermatitis and blistering, irritant keratoconjunctivitis, blindness, nausea and vomiting. Many plants in the family of *Euphorbiaceae* contain euphorbon (a dangerous poison) [22, 23].

*Euphorbia abyssinica* is used for live fencing, firewood, and timber. There is no ethnobotanical data on the use of *Euphorbia abyssinica* as a traditional medicinal plant outside Ethiopia. It is rather classified as a poisonous plant by the Food and Drug Administration of the United States of America [24]. In Ethiopian traditional medical practices, the plant has been utilized for treatment of various diseases including malaria [25–31]. Its medicinal effects are mainly confined to its latex and roots [14, 22]. The mode of preparation of the plant for treatment of malaria by local people and/or herbalists was as followed: the fresh root was collected; it was chopped using stone and woody materials; the chopped plant material was dried under sunlight; the dried product was further powdered and eaten with egg then a lot of milk was drunk.

However, the safety, quality and efficacy of *Euphorbia abyssinica* for treatment of malaria have not been verified scientifically. There are reports on the anti-malarial activities of two other related species namely *Euphorbia hirta* [32] and *Euphorbia prostrata* [33]. It is justifiable to scientifically evaluate its anti-malarial activity in rodent malarial model to support or deject its folk use in the treatment of malaria. This study was, therefore, designed to evaluate the anti-malarial activity of 80% methanolic root extract of *Euphorbia abyssinica* against *Plasmodium berghei* infection in mice.

## Methods

### Collection and preparation of the plant material

The fresh roots of *E. abyssinica* were collected in Nekemte town, East Wollega zone, Western Ethiopia in March 2018. The plant was identified and authenticated by botanists in Department of Biology, Wollega University, Ethiopia. A voucher specimen was deposited at National Herbarium, Addis Ababa University, Ethiopia. The collected roots were cleaned, washed, and air dried under shade at room temperature. The dried roots were coarsely powdered. Then the powdered plant materials were stored in a plastic container and they were kept at room temperature until extraction.

### Extraction of the plant materials

The powdered samples of roots of *Euphorbia abyssinica* (355 g) were weighed by sensitive electrical balance (Adam equipment, France). They were extracted by maceration with 80% methanol (Okhla Industrial, India) for three consecutive days with orbital shaker (Lab Teck, India) in Chemistry Laboratory of Department of Chemistry, Wollega University. After 3 days, the mixture was filtered with Whatman filter paper number one (Whatman, England). The residue was re-macerated twice for the same duration of days and then the mixture was filtered. The combined filtrates were dried by rota evaporator (Rotovap, India) and hot oven (250 V, France). The weight of the dried extract was measured to determine the percentage yield. The dried extract was kept in a fridge until experiment.

### Experimental animals

Twenty-five male Swiss albino mice inbred in the Animal House of Ethiopian Public Health Institute were used for anti-malarial activity testing. Five female Swiss albino mice were used for acute toxicity study. They were housed in plastic cages with softwood shavings and chips as beddings. They had free access to pellet diet and clean drinking water. All animals were acclimatized to the working environment 1 week before the beginning of the experiment.

### Parasites

Chloroquine-sensitive *Plasmodium berghei* (ANKA strain) was used for induction of malaria in experimental mice. Mice previously infected with *P. berghei* were used as donor. The donor *P. berghei* infected mice were obtained from Traditional Medicine Department of Ethiopian Public Health Institute. The parasites were subsequently maintained in laboratory by serial passage of blood from donor infected mice to naive one via intra-peritoneal route on weekly basis.

### Study design and sampling method

Experimental study design was used. Simple random sampling technique was employed for grouping of experimental animals and assignments of treatments.

### Inoculum preparation

The parasitaemia of the previously *P. berghei* infected donor mouse was determined. The blood from these mice was collected via cardiac puncture with a parasitaemia of 35% into a test tube having 0.5% trisodium citrate. The blood was then diluted with normal saline to give 2 × 10^7^ infected red blood cells (RBCs) in an injection volume of 0.2 ml. Each mouse was then infected by injecting 0.2 ml of this diluted blood via intra-peritoneal route which contained 2 × 10^7^ infected RBCs [34].

### Grouping and dosing of animals

Twenty-five mice were grouped into five groups of five mice each. Group I mice was treated with the vehicle (distilled water, 10 ml/kg, which served as negative control), group II, III and IV mice were treated with 200, 400, and 600 mg/kg of crude extract, respectively and the last group (group V) mice was treated with the standard drug [chloroquine (Addis Pharmaceuticals Factory, Ethiopia), 25 mg/kg, which served as positive control]. Since patients used the plant traditionally for treatment of malaria orally, all treatments were administered to mice via intra-gastric route using oral gavage for safe ingestion.

### In vivo acute toxicity test

Acute toxicity test of the extract was conducted in five nulliparous, non-pregnant female Swiss albino mice from 8 to 12 weeks old. They were fasted from food overnight. They were dosed sequentially with the extract at a limit dose of 2000 mg/kg. If the first animal was survived, the four additional animals were dosed sequentially so that a total of five animals were tested. Accordingly, mice were observed for any sign of gross physical and behavioural changes over an hour, 4 h, 24 h, and for 14 days. If three or more animals were survived, the median lethal dose (LD_50_) will be greater than the test dose. At the end, surviving animals were humanely killed. The test procedure was done according to Organization for Economic Cooperation and Development (OECD) guideline [35].

### Four-day chemosuppressive anti-malarial activity test

The chemosuppressive test was done by using a standard 4-day suppressive test against *P. berghei* infection in mice as described by Fidock et al. [36]. After standard parasite inoculation, 25 mice were randomly divided into five groups of five mice each. Treatments were started 3 h after infection for each group accordingly and then continued for three consecutive days (from D_0_ to D_3_). On the fifth day (D_4_), thin blood films were made from the tail of each mouse on a microscopic slide (Sciencelab, USA).

### Peripheral blood smear preparation

Thin blood smears were made from the tail of each mouse on the fifth day (D_4_). The smears were applied on microscopic slides and the blood was drawn evenly across a second slide to make a thin blood film and allowed to dry at room temperature. They were fixed with absolute methanol and stained with 10% Giemsa stain (Macsenlab, India) at pH 7.2 for 15 min. Each stained slide for each mouse was examined under microscope (Olympus, Japan).

### Parasitaemia determination

The parasitaemia level was determined by counting the number of parasitized RBCs in random fields of the microscope. The smears were read and counted by a laboratory technician to make the reader blind to the category. Average parasitaemia and percent parasitaemia suppression were calculated using the following formula [36].

$$ \% \;{\text{Parasitaemia}} = \frac{{{\text{Number}}\;{\text{of}}\;{\text{Infected}}\;{\text{RBCs}}}}{{{\text{Total}}\;{\text{Number}}\;{\text{of}}\;{\text{RBCs}}}} \times 100 $$

$$ \% \;{\text{Suppression}} = \frac{{\left( {{\text{Mean}}\;{\text{Parasitaemia}}\;{\text{of}}\;{\text{NC}} - {\text{Mean}}\;{\text{Parasitaemia}}\;{\text{of}}\;{\text{TG}}} \right)}}{{{\text{Mean}}\;{\text{Parasitaemia}}\;{\text{of}}\;{\text{NC}}}} \times 100 $$

where NC, negative control; TG, treated group.

### Determination of mean survival times

Mean survival time (MST) is another parameter that is commonly used to evaluate the efficacy of anti-malarial plant extracts. An extract that results in survival time greater than that of infected non-treated mice was considered as active. Death occurring before day 5 of infected and treated mice was regarded as toxic death. Mortality was monitored daily. The number of the days from the time of inoculation of the parasite up to death was recorded for each mouse in the treatment and control groups throughout the follow up period. The survival time for each mouse was recorded after the treatment periods.

$$ {\text{MST}} = \frac{{{\text{Sum}}\;{\text{of}}\;{\text{Survival}}\;{\text{Time}}\;{\text{of}}\;{\text{All}}\;{\text{Mice}}\;{\text{in}}\;{\text{a}}\;{\text{Group}}\,\left( {\text{days}} \right)}}{{{\text{Total}}\;{\text{Number}}\;{\text{of}}\;{\text{Mice}}\;{\text{in}}\;{\text{That}}\;{\text{Group}}}} $$

### Body weight determination

Similarly, body weight loss is one feature of rodent malaria infections. Body weight of each mouse was measured to determine the effectiveness of the extract. The body weight of each mouse in all groups was taken before infection (D_0_) and after treatment (D_4_). The weight of each mouse was measured using sensitive electrical balance. The average body weight changes of extract treated groups were compared with the control groups. The average body weight change of each treatment group was calculated using the following formula.

$$ \begin{aligned} {\text{Average}}\;{\text{Weight}}\;{\text{Change}} = {\text{Average}}\;{\text{D}}_{4} \;{\text{Weight}}\;{\text{of}}\;{\text{a}}\;{\text{Group}} \hfill \\ - {\text{Average}}\;{\text{D}}_{0} \;{\text{Weight}}\;{\text{of}}\;{\text{that}}\;{\text{Group}} \hfill \\ \end{aligned} $$

### Statistical analysis

Results of the study were expressed as mean ± SEM (standard error of mean) for each treatment group. Data on levels of parasitaemia, changes in body weights and survival times were analysed using windows SPSS version 20. One-way ANOVA was used to analyse differences among groups. Subgroup analysis was done using Tukey post hoc test. The difference was considered statistically significant if P-value < 0.05.

### Data quality control

Randomization was used during grouping of experimental animals and assignments of treatments. Codes were utilized for all microscopic slides. Parasitized RBCs were counted blindly by a laboratory technician.

### Ethical consideration

Ethical clearance was requested and approved by the ethical committee of College of Health Sciences, Wollega University, Ethiopia. During experimental procedures, experimental animals were handled and cared according to the internationally accepted laboratory animals’ use, care and welfare guideline [37].

## Results

### Percentage yield of the crude plant extract

The percentage yield of 80% methanolic crude extract of the roots of *Euphorbia abyssinica* was 7.04% weight by weight. Its actual yield was 25 g. The dry extract was dark red and hygroscopic.

### In vivo acute toxicity of the crude plant extract

All mice tested at the limit dose of 2000 mg/kg were normal during the observation periods. Gross physical and behavioural changes including rigidity, sleep, diarrhoea, depression, abnormal secretion and hair erection were not observed within 24 h. There were no deaths occurred within 14 days. The LD_50_ might be greater than the limit dose. The mice were humanly killed after the observation periods. This safety data was used for selection of the three dose levels (200, 400 and 600 mg/kg) of the crude root extract of *Euphorbia abyssinica* for the anti-malarial activity evaluation in mice.

### Four-day chemosuppressive anti-malarial activity of the crude plant extract

The 80% methanolic crude extract of the roots of *Euphorbia abyssinica* showed significant chemosuppressive anti-malarial activity against *P. berghei* infection in mice. Mice treated with 200, 400 and 600 mg/kg extract showed 66.87%, 84.94% and 93.69% chemosuppressive anti-malarial activities, respectively, as compared to distilled water treated mice. Similarly, 400 and 600 mg/kg extract treated mice showed significant chemosuppressive anti-malarial activity variations as compared to 200 mg/kg extract treated mice as shown in Table 1.

**Table 1:** Four-day chemosuppressive anti-malarial activity of 80% methanolic crude extract of the roots of *Euphorbia abyssinica* against *Plasmodium berghei* infection in mice

Treatments	Doses	Parasitaemia (%)	Chemosuppression (%)
Distilled water	10 ml/kg	24.90 ± 1.54	0.00
*Euphorbia abyssinica* crude root extract	200 mg/kg	8.25 ± 0.56	66.87^a1,c2,d1,e1^
	400 mg/kg	3.75 ± 0.75	84.94^a1,b2,e3^
	600 mg/kg	1.57 ± 0.49	93.69^a1,b1^
Chloroquine	25 mg/kg	0.60 ± 0.38	97.59^a1,b1,c3^

Values are expressed as mean ± SEM; n = 5

^1^P < 0.001; ^2^P < 0.01; ^3^P < 0.05

^a^As compared to negative control

^b^As compared to 200 mg/kg extract

^c^As compared to 400 mg/kg extract

^d^As compared to 600 mg/kg extract

^e^As compared to positive control

### Effect of crude plant extract on survival times of mice

Distilled water treated mice died within a week of infection while chloroquine-treated group survived and were cured. Mice treated with 600 mg/kg extract significantly survived longer than the corresponding distilled water treated mice (P < 0.001) and 200 mg/kg extract treated mice (P < 0.05) as shown in Table 2.

**Table 2:** Effect of 80% methanolic crude extract of the roots of *Euphorbia abyssinica* on the mean survival times of *Plasmodium berghei* infected mice

Treatments	Doses	Mean survival times (days)
Distilled water	10 ml/kg	7.40 ± 0.51
*Euphorbia abyssinica* crude root extract	200 mg/kg	9.00 ± 0.71^d2,e1^
	400 mg/kg	9.80 ± 0.80^e1^
	600 mg/kg	12.00 ± 0.71^a1,b2,e2^
Chloroquine	25 mg/kg	15.00 ± 0.00^a1,b1,c1,d2^

Values are expressed as mean ± SEM; n = 5

^1^P < 0.001; ^2^P < 0.05

^a^As compared to negative control

^b^As compared to 200 mg/kg extract

^c^As compared to 400 mg/kg extract

^d^As compared to 600 mg/kg extract

^e^As compared to positive control

### Effect of crude extract on body weights of mice

Although the differences were not statistically significant, all extract treated mice prevented body weight loss as compared to distilled water treated mice. There were no significant differences among the body weight gains of extract treated groups. Chloroquine-treated mice did not lose weight as shown in Table 3.

**Table 3:** Effect of 80% methanolic crude extract of the roots of *Euphorbia abyssinica* on the body weights of *Plasmodium berghei* infected mice

Treatments	Doses	Body weight (g)		
		D_0_-weight	D_4_-weight	Weight change
Distilled water	10 ml/kg	40.00 ± 2.64	36.34 ± 2.40	− 3.66
*Euphorbia abyssinica* crude root extract	200 mg/kg	37.72 ± 0.78	34.30 ± 1.70	− 3.42^c1^
	400 mg/kg	43.76 ± 1.83	42.78 ± 1.87	− 0.98
	600 mg/kg	41.92 ± 1.07	40.40 ± 1.17	− 1.52
Chloroquine	25 mg/kg	40.26 ± 0.80	41.38 ± 0.87	1.12^a1,b1^

Values are expressed as mean ± SEM; n = 5

^1^P < 0.01

^a^As compared to negative control

^b^As compared to 200 mg/kg extract

^c^As compared to positive control

## Discussion

More than 80% of African population such as Ethiopian’s used plant-derived drugs to meet their primary healthcare demands. Three-fourth of Ethiopian lands is malarious and 68% population lived in such malaria risky areas. Ethiopia is among the malaria endemic and poorest country in the world. Because of cultural entrenchment, cheapness and easily accessibility, medicinal plants are the main sources of drug for different health ailments including malaria in Ethiopian traditional medical practices. More than 200 medicinal plants are currently utilized for treatment of malaria in Ethiopia. However, the safety, quality and efficacy of these medicinal plants are largely unknown. Assessments of these qualities of medicinal plants are among the prioritized issues in the country [38–41].

Rodent malaria model in mice is the most extensively used for the primary in vivo investigation of natural and synthetic anti-malarial agents. This model can detect prodrugs that require metabolic reactivation and the effect of agents on immune systems. The 4-day chemosuppressive model is the most widely used model for preliminary assessment of new anti-malarial agents [42].

All *Euphorbia abyssinica* extract treated mice showed significant chemosuppressive anti-malarial activities (P < 0.001) as compared to distilled water treated mice in a dose-dependent manner. Mice treated with 400 and 600 mg/kg extract had significant chemosuppressive anti-malarial activities (P < 0.01 and P < 0.001, respectively) as compared to 200 mg/kg extract treated mice. A new anti-malarial compound is considered active when it causes parasitaemia suppression 30% or more [42]. This supports the anti-malarial activity of the current plant material. Similar dose-dependent anti-malarial activities were reported by many plant extracts including *Brassica nigra* [43] and *Indigofera spicata* [44] in Ethiopia.

The current findings were further supported by the positive anti-malarial activities of two closely related plant species, namely *Euphorbia hirta* [32] and *Euphorbia prostrate* [33]. The crude methanolic root extract of the current plant (200, 400, 600 mg/kg) showed very significant (P < 0.001) chemosuppressive anti-malarial activities of 66.87% to 93.69% while the crude ethanol extract of *Euphorbia hirta* whole plant (200, 400 and 800 mg/kg) showed significant (P < 0.05) chemosuppressive anti-malarial activity of 51% to 59%, respectively against *P. berghei* infection in mice when compared to vehicle treated mice. Both *Euphorbia hirta* and *Euphorbia prostrata* had significant in vitro anti-plasmodial activities against *P. falciparum*. The current anti-malarial activity of the plant could be shared with the other pharmacological activities including acaricides and tick repellant [45], worm expelling [46], antibacterial [21, 47], antifungal [47], and wound healing [46, 48] activities of the plant. According to classification of new in vivo anti-malarial activity of plant extracts [49], the methanolic crude extract of roots of *Euphorbia abyssinica* showed very good 4-day chemosuppressive anti-malarial activity.

Survival time is another parameter that evaluates the anti-malarial activity of plant extracts. An extract that resulted in survival time greater than that of infected non-treated mice was considered as active [36, 42]. Mice treated with 600 mg/kg of extract significantly survived longer than distilled water treated mice. This might explain the anti-malarial activity of the plant extract. However, the mean survival times of extract treated mice were shorter than chloroquine-treated ones. This might be the fast reversible action or rapid elimination phase of the extract. This was in line with other experimental reports [43, 44, 50]. Chloroquine-treated mice survived and were cured.

Body weight loss is another feature of *P. berghei* infected mice. This is resulted from the appetite depressant action on mice, the disturbed metabolic function and hypoglycaemic effect of the parasite [36]. The weight changes caused by all extract treated mice were not significant compared to distilled water treated mice. The difference might be explained by the imbalance of the protective effect of the extract and the cumulative pathologic changes associated with the infection. A similar finding was reported by another experimental study [44].

The anti-malarial activities of plants are due to the presence of bioactive secondary metabolites in the crude plant material. Different secondary metabolites including alkaloids, glycosides, indoles, phenols, tannins, saponins and steroids [21, 48] have been reported from the extract of *Euphorbia abyssinica*. These metabolites have established anti-malarial activities [51]. Their anti-malarial activities could have resulted from single or in synergistic action [52]. The possible anti-malarial activity of the plant might be through anti-oxidation and free radical scavenging, immunomodulatory, intercalation in deoxyribonucleic acid (DNA), inhibition of protein synthesis, interference with enterocytes’ invasion, or by any other unknown mechanisms.

## Conclusion

From this study, it can be concluded that the root of *Euphorbia abyssinica* showed very good 4-day chemosuppressive anti-malarial activity. The plant may contain biologically active principles which are relevant for treatment of malaria. In addition, the extract did not show any sign of toxicity and death at limit dose of 2000 mg/kg. Further phytochemical, toxicological and pharmacological investigations are, therefore, required to evaluate its full anti-malarial potential.

## Data Availability

The datasets used and/or analysed during the current study are available from the corresponding author on reasonable request.

## Abbreviations

ANOVA: analysis of variance
DNA: deoxyribonucleic acid
IC_50_: median inhibitory concentration
LD_50_: median lethal dose
MST: mean survival time
OECD: Organization for Economic Cooperation and Development
RBCs: red blood cells
SEM: standard error of mean
SPSS: statistical package for social sciences
WHO: World Health Organization
